# Anesthetic Management of Eosinophilic Granulomatosis with Polyangiitis: A Narrative Review with an Illustrative Case in Cardiac Surgery

**DOI:** 10.3390/jpm16050241

**Published:** 2026-04-30

**Authors:** Debora Emanuela Torre, Carmelo Pirri

**Affiliations:** 1 Department of Cardiac Anesthesia and Intensive Care Unit, Cardiac Surgery, Ospedale dell’Angelo, Mestre, 30174 Venice, Italy; 2 Department of Neurosciences, Institute of Human Anatomy, University of Padova, 35121 Padua, Italy; carmelo.pirri@unipd.it

**Keywords:** eosinophilic granulomatosis with polyangiitis, anesthetic management, Churg–Strauss, EGPA anesthetic management, Churg–Strauss anesthetic management

## Abstract

**Background**: Eosinophilic granulomatosis with polyangiitis (EGPA), formerly Churg–Strauss syndrome, is a rare necrotizing vasculitis characterized by asthma, eosinophilia, and systemic granulomatosis vasculitis. Perioperative risk is primarily driven by airway hyperreactivity, potential cardiac disease, chronic immunosuppressive therapy, and reported alterations in plasma cholinesterase activity. Evidence specifically addressing anesthetic management remains scarce and largely limited to case-based reports. **Methods**: A focused narrative review was conducted by searching MEDLINE (via PubMed), Scopus, and Embase from inception to January 2026 for publications reporting perioperative anesthetic management in patients with EGPA/Churg–Strauss syndrome. Case reports and case-based descriptions providing explicit anesthetic details were qualitatively synthesized. **Results**: Available evidence consists predominantly of isolated case reports across heterogeneous surgical settings, including ENT, abdominal, orthopedic, ambulatory, pediatric, and rare cardiac procedures. Recurring perioperative principles include optimization of bronchial disease and continuation of inhaled therapy; minimization of airway stimulation and avoidance of histamine-releasing drugs; selection of induction agents preserving hemodynamic stability in the presence of myocardial involvement; preference for non-depolarizing neuromuscular blockade with quantitative monitoring (and consideration for sugammadex when appropriate); individualized corticosteroid management and multimodal, opioid-sparing analgesia, often supported by regional techniques. **Conclusions**: In the absence of dedicated perioperative guidelines, anesthetic care for EGPA should be individualized based on clinical phenotype and organ involvement. A structured approach targeting airway protection, cardiovascular stability, safe neuromuscular management, and opioid-sparing analgesia may represent a pragmatic risk-mitigation framework. These considerations are illustrated by an institutional experience in mitral valve surgery.

## 1. Introduction

Eosinophilic granulomatosis with polyangiitis (EGPA) is a chronic inflammatory disease characterized by eosinophilic-rich granulomatosis inflammation and necrotizing vasculitis of small-to-medium-sized vessels [1,2]. Typical clinical features include asthma, chronic rhinosinusitis, pulmonary infiltrates, peripheral eosinophilia, and systemic organ involvement. Although the respiratory tract is most frequently involved, EGPA is a systemic disease that may affect multiple organs, including the heart, peripheral nervous system, kidneys, and gastrointestinal tract, with significant implications for morbidity and mortality [3,4]. Cardiac involvement represents one of the most severe manifestations of EGPA and constitutes a major determinant of prognosis. Myocarditis, cardiomyopathy, coronary arteritis, and valvular diseases have all been described and may substantially increase perioperative risk, particularly in the setting of major surgery [3,4]. From an anesthetic perspective, the coexistence of airway hyperreactivity, potential myocardial dysfunction, and chronic immunosuppressive therapy poses unique challenges. Despite these relevant implications, the literature focusing on anesthetic management remains limited, consisting mainly of isolated case reports, with no standardized perioperative guidelines available. Recommendations are often extrapolated from general principles of asthma management, systemic vasculitis care, and expert opinion [5,6,7]. Consequently, anesthetic decision-making in this population is frequently based on individualized clinical judgment rather than on standardized protocols. From the perspective of personalized medicine, EGPA represents a paradigmatic condition in which a “one-size-fits-all” anesthetic strategy is inadequate. The marked heterogeneity of organ involvement, variability in disease activity, and differences in chronic therapies generate highly individualized perioperative risk profiles. Consequently, anesthetic management may be tailored to the specific biological and clinical characteristics of each patient, integrating pulmonary function, cardiovascular status, immunologic background, and previous treatment exposure. Framing perioperative decision-making within a personalized medicine approach is therefore essential to optimize safety and outcomes in this complex population. The aim of the present narrative review is to summarize current knowledge regarding anesthetic and perioperative management in patients with EGPA, with particular focus on airway protection, neuromuscular blockade, hemodynamic management, corticosteroid therapy, and opioid-sparing analgesic strategies.

## 2. Literature Review

### 2.1. Methods

**Figure 1:**
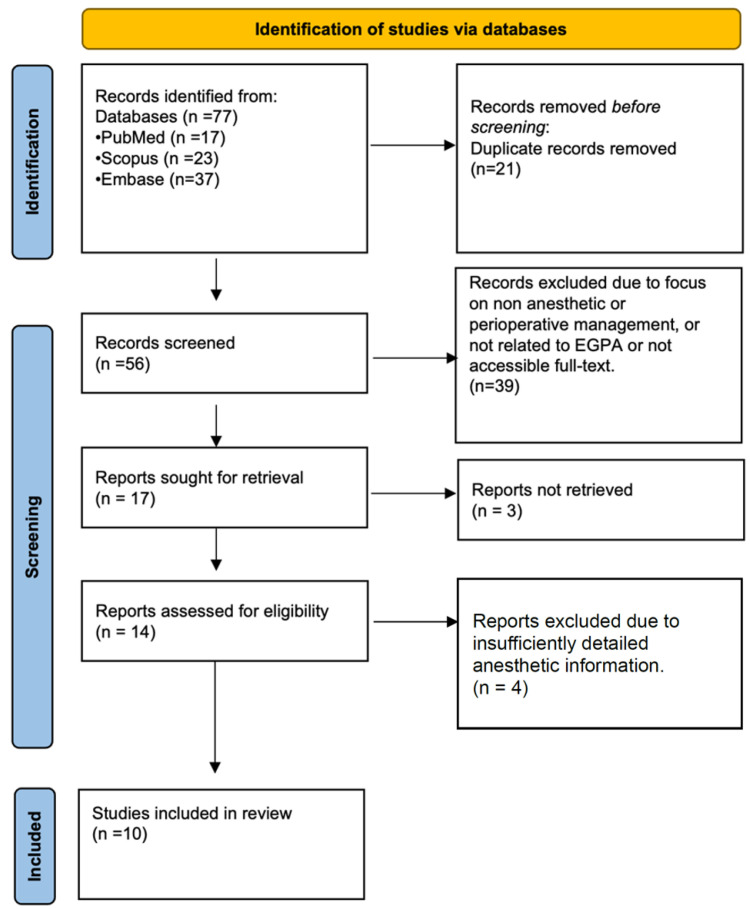
Study selection process for case-based descriptive synthesis.

This manuscript is structured as a focused narrative review. A supportive literature search was performed to identify published case reports describing anesthetic and perioperative management in patients with eosinophilic granulomatosis with polyangiitis (EGPA). The search was intended to contextualize clinical practice rather than to provide an exhaustive systematic synthesis. MEDLINE (via PubMed), Scopus, and Embase were searched from database inception to 12 January 2026. The search combined disease-specific terms and perioperative terms using the following strategy: (“Churg–Strauss syndrome” or “eosinophilic granulomatosis with polyangiitis”) and (anesthesia or anesthesia). Search terms were adapted as appropriate for each database. No language restrictions were applied. A total of 77 records were identified, of which 56 remained after duplicate removal. Inclusion criteria were: original reports describing anesthetic or perioperative management in patients with EGPA/Churg-Struss syndrome and availability of explicit anesthetic details. Review articles without primary case data and studies lacking perioperative information were excluded. Titles and abstracts were screened by the authors to identify potentially relevant reports. Ten case reports met the inclusion criteria and were included in the qualitative synthesis (Figure 1). Given the narrative design and the exclusive presence of low-level evidence (isolated case reports), no formal risk-of-bias assessment was performed. Findings were synthesized descriptively without meta-analysis. In addition to the database search aimed at identifying case reports for qualitative synthesis, selected high-quality reviews, consensus statements, and clinical guidelines were consulted to provide a pathophysiological context and to support general anesthetic principles discussed in the manuscript. These sources were not part of the case-based inclusion analysis but were used to contextualize and interpret the available case literature.

### 2.2. Results

The literature search identified a limited number of publications specifically addressing anesthetic and perioperative management in patients with EGPA. After screening titles, abstracts, and full-texts, a total of ten case reports providing explicit anesthetic or perioperative details were considered eligible for inclusion. No prospective studies, cohort analyses, or formal guidelines were identified. The available reports encompassed a wide spectrum of surgical contexts, including otolaryngologic, abdominal, orthopedic, ambulatory, pediatric, and cardiac procedures (Table 1). All included publications consisted of single-patient case reports, reflecting the rarity of the condition and the absence of higher-level evidence. Asthma or clinically significant airway hyperreactivity was explicitly reported in the majority of cases (7/10), frequently accompanied by chronic rhinosinusitis or recurrent respiratory infections. Cardiovascular involvement was described in one case, including one patient undergoing major cardiac surgery and another with significant coronary artery pathology. General anesthesia was employed in most reports (7/10), whereas regional or neuraxial techniques were used in three cases, primarily to minimize airway manipulation in patients with severe pulmonary involvement. Rocuronium was the most commonly reported non-depolarizing neuromuscular blocking agent when specified. Sugammadex reversal was explicitly described in only one case. Detailed neuromuscular monitoring strategies were inconsistently reported across studies. A severe peri-induction anaphylactic reaction was documented in one case, representing the initial presentation of EGPA. Another report highlighted prolonged neuromuscular blockade associated with reduced plasma cholinesterase activity following succinylcholine administration, underscoring a potential disease-specific pharmacologic consideration.

Despite the heterogeneity of clinical scenarios, the reports consistently highlighted recurrent perioperative priorities, particularly careful airway management, cautious selection of anesthetic agents, individualized neuromuscular blockade strategies, and opioid-sparing analgesia. Collectively, the available literature underscores the need for a personalized, phenotype-driven approach to anesthetic care in patients with EGPA.

## 3. Discussion

### 3.1. Background

EGPA typically progresses through overlapping allergic, hypereosinophilic, and vasculitic phases. Diagnosis is based on a constellation of clinical and laboratory findings; no single pathognomonic test exists. Key features include asthma, peripheral eosinophilia, neuropathy, pulmonary infiltrates, and sinonasal abnormalities as defined by the American College of Rheumatology (ACR) classification criteria [1,2,4,16].

EGPA is a heterogeneous systemic vasculitis that may present with overlapping allergic, eosinophilic, and vasculitic manifestations. Diagnosis relies on the integration of clinical features, laboratory findings, and, when available, histopathology, as no single pathognomonic test exists. In 2022, the American College of Rheumatology (ACR) and the European Alliance of Associations for Rheumatology (EULAR) published a validated classification based on weighted items (e.g., maximum eosinophil count ≥ 1 × 10^9^/L, obstructive airway disease, nasal polyps, extravascular eosinophilic-predominant inflammation on biopsy, and peripheral neuropathy features), with negative weights for c-ANCA/anti-PR3 positivity and hematuria. These criteria capture the biological and phenotypic heterogeneity of EGPA and provide a structured framework for contemporary classification in research settings [1,2,4,16,17].

These criteria reinforce the distinction between eosinophil-predominant and vasculitic phenotypes and provide a more structured approach to disease classification.

Anesthetic risk in EGPA is dominated by three main factors: airway hyperreactivity secondary to eosinophilic asthma, potential cardiac involvement, chronic immunosuppressive therapy, and possible cholinesterase deficiency [2,4,9]. Because dedicated perioperative guidelines are lacking, anesthetic strategies may be individualized (Table 2).

### 3.2. Pulmonary Optimization and Airway Management

Asthma and airway hyperresponsiveness are nearly universal in EGPA and constitute the principal anesthetic challenge [16,17,18]. Bronchospasm may be triggered by airway manipulation, histamine-releasing drugs, or inadequate anesthetic depth [18]. Preoperative pulmonary evaluation, continuation of inhaled bronchodilators, and avoidance of respiratory irritants are recommended [6,7,19,20].

Published case reports describe successful perioperative airway management in patients with EGPA undergoing a variety of surgical procedures, including endoscopic sinus surgery, laparoscopic abdominal surgery, oncologic surgery, ambulatory procedures, and pediatric thoracoscopic interventions. In these reports, pre-induction strategies commonly included optimization of pulmonary status, continuation of inhaled bronchodilators, avoidance of airway irritation, the use of bronchodilatory or airway-stabilizing agents such as antihistamines, magnesium sulfate, benzodiazepines, topical lidocaine, and ketamine to attenuate reflex bronchoconstriction, as well as the preferential use of videolaryngoscopy to minimize airway manipulation during tracheal intubation [6,21,22,23,24].

### 3.3. Induction and Maintenance of Anesthesia

Induction requires preservation of hemodynamic stability while preventing bronchospasm. Etomidate is preferred in patients with impaired myocardial reserve due to minimal cardiovascular depression [23]. Synthetic opioids such as fentanyl or sufentanil are recommended because they do not include histamine release, in contrast to morphine, which may trigger bronchoconstriction and vasodilation [6,25,26,27]. Remifentanil, due to its rapid onset and ultra-short context-sensitive half-life, may represent a useful intraoperative option in patients with EGPA, allowing precise titration of sympathetic and airway responses while minimizing residual postoperative respiratory depression. Its use should be balanced within a multimodal analgesic strategy aimed at reducing cumulative opioid exposure, particularly in the postoperative period [28].

Volatile anesthetics, particularly sevoflurane, exert bronchodilatory properties and reduce airway resistance through direct smooth muscle relaxation, effects that have been utilized in the management of severe bronchospasm and status asthmaticus [29]. Experimental studies have demonstrated that sevoflurane significantly decreases respiratory resistance and increases dynamic compliance [30]. Among volatiles, sevoflurane is often preferred in patients with reactive airway disease due to its minimal airway irritation [29]. Balanced anesthesia with sevoflurane and short-acting opioids has been widely adopted in patients with asthma and has been successfully applied in a published case report of EGPA across different surgical settings, without major respiratory complications. In particular, sevoflurane-based techniques combined with short-acting opioids have been reported during ENT, abdominal, oncologic, ambulatory, and pediatric procedures, supporting the use of balanced anesthesia tailored to airway reactivity and cardiovascular status in this population [10,11,31].

### 3.4. Neuromuscular Blockade and Cholinesterase Deficiency

Reports of reduced plasma cholinesterase activity in EGPA are limited to isolated case descriptions and do not allow reliable estimation of its prevalence. Therefore, categorical avoidance of succinylcholine cannot be supported by robust evidence. However, given the potential for prolonged neuromuscular blockade described in individual reports, a cautious and individualized approach is warranted. Both during rapid sequence induction and in routine anesthetic practice, the use of non-depolarizing neuromuscular blocking agents (e.g., rocuronium) represents a pragmatic strategy and should systematically include quantitative neuromuscular monitoring to ensure appropriate depth of blockade and safe recovery. A predefined reversal plan (such as sugammadex when appropriate) should also be considered. In selected cases, preoperative pseudocholinesterase testing may be considered if clinical suspicion exists, although routine screening cannot currently be recommended based on the available evidence [9,12,18,32,33,34]

### 3.5. Cardiovascular Considerations

Cardiac involvement in EGPA represents a major determinant of perioperative risk and disease-related mortality [35].

Myocarditis, cardiomyopathy, coronary arteritis, pericardial disease, and valvular dysfunction may all occur, often in the absence of overt symptoms [3,4,36,37].

For this reason, comprehensive preoperative cardiovascular assessment, including echocardiography and, when indicated, advanced imaging, may play an important role in defining ventricular function and guide anesthetic planning [38,39]. From an anesthetic standpoint, patients with EGPA-related cardiac disease require careful selection and titration of induction and maintenance agents to avoid hemodynamic instability. Agents with minimal negative inotropic effects are generally preferred, and invasive monitoring may be necessary in major surgical procedures. The literature consistently emphasizes that perioperative management should be individualized according to the specific pattern and severity of cardiac involvement.

### 3.6. Perioperative Corticosteroid Management

Chronic corticosteroid therapy is a cornerstone of EGPA treatment, and many patients present for surgery while receiving long-term glucocorticoids. Traditional practice has often advocated routine administration of “stress-dose” steroids in the perioperative period. However, current evidence indicates that traditional supplementation is generally unnecessary in patients maintained on low-dose regimens (typically <10–15 mg/day of prednisone or equivalent) who do not exhibit clinical features of hypothalamic–pituitary–adrenal axis suppression [40,41]. Perioperative steroid management should therefore be individualized, taking into account baseline corticosteroid dose, duration of therapy, degree of surgical stress, and clinical indicators of adrenal suppression. Patients receiving chronic moderate-to-high-dose corticosteroids may require perioperative supplementation according to institutional endocrine protocols, whereas those on low-maintenance doses without evidence of hypothalamic–pituitary–adrenal axis impairment may not universally require additional dosing. Initiation of perioperative corticosteroid therapy in patients not previously receiving glucocorticoids should not be routine and may be based on evidence of active EGPA or risk of adrenal insufficiency, ideally in coordination with the treating rheumatologist or internist [42,43,44,45].

Close interdisciplinary collaboration with treating physicians may be appropriate to ensure continuity of immunosuppressive therapy while minimizing the risk of adrenal insufficiency or infection.

### 3.7. Postoperative Analgesia

Effective postoperative analgesia represents a crucial component of perioperative care in patients with EGPA, particularly in the presence of severe asthma or pulmonary involvement. Nonsteroidal anti-inflammatory drugs (NSAIDs) are often avoided because of the potential risk of bronchospasm and hypersensitivity reactions. Morphine and other histamine-releasing opioids may similarly exacerbate bronchial reactivity and are therefore generally discouraged [25,26,27]. Multimodal and opioid-sparing strategies are widely advocated in the literature. Regional and neuraxial techniques, when feasible, provide effective analgesia while minimizing systemic opioid exposure and associated respiratory depression. The growing availability of ultrasound-guided fascial plane blocks has further expanded the options for targeted postoperative pain control in this population. Overall, individualized analgesic planning tailored to respiratory status and surgical context may play an important role in optimizing recovery [10,13,15,46,47].

### 3.8. Illustrative Case Experience

To contextualize and operationalize the perioperative principles emerging from the reviewed literature, we present an institutional case illustrating phenotype-driven anesthetic decision-making in a patient with EGPA undergoing major cardiac surgery. The case is presented as an illustrative application of the principles synthesized from the available literature and is not intended to provide generalizable evidence. Rather, it serves to operationalize phenotype-driven decision-making in a complex real-world perioperative scenario. The case is described with clinically relevant perioperative details to demonstrate how airway hyperreactivity, cardiac involvement, and chronic corticosteroid therapy influenced anesthetic planning and intraoperative management. Ethical review and approval were waived for this study by the Territorial Ethics Committee of the Central-East Veneto Area due to representing a retrospective description of a single clinical case and not involving any experimental intervention. Written informed consent for publication was obtained from the patient. All clinical images were fully de-identified prior to submission in accordance with institutional data protection standards.

#### 3.8.1. Preoperative Assessment

A 64-year-old man was admitted for severe mitral regurgitation with markedly reduced left ventricular ejection fraction (32%) and underwent mitral valve replacement via median sternotomy (St. Jude Epic 31 bioprosthesis). His medical history included hypertension, chronic atrial fibrillation, diabetes mellitus, and established eosinophilic granulomatosis with polyangiitis treated with prednisone 10 mg/day and budesonide-formoterol inhalational therapy. EGPA manifestations included marked peripheral eosinophilia (8,500 cells/μL; 42% of leukocytes), asthma with recurrent bronchospastic episodes, chronic rhinosinusitis, sensory neuropathy of the left lower limb, and pulmonary infiltrates on chest radiography. Preoperative pulmonary function tests demonstrated a moderate obstructive ventilatory defect (forced expiratory volume in one second, FEV_1_ 58% predicted, forced vital capacity, FVC 85% predicted, and FEV_1_/FVC ratio 68%). Cardiac magnetic resonance imaging performed four months earlier revealed late gadolinium enhancement of the inferobasal mid-ventricular wall, consistent with prior myocardial involvement. Arterial blood gas analysis on room air showed mild hypoxemia (PaO_2_ 73 mmHg) and slight hypercapnia (PaCO_2_ 46 mmHg), consistent with the underlying obstructive ventilatory defect.

#### 3.8.2. Induction and Airway Management

Premedication consisted of intravenous chlorphenamine 10 mg, midazolam 1 mg, and magnesium sulfate 1 g to reduce airway irritability and mitigate bronchospastic responses. Topical lidocaine spray was applied to the tongue, oropharynx, and vocal cords prior to laryngoscopy. Intravenous ketamine 30 mg and lidocaine 1 mg/kg were administered to attenuate reflex bronchoconstriction and reduce intraoperative opioid requirements. Anesthesia was induced with etomidate 0.2 mg/kg, sufentanil 0.3 µg/kg, and rocuronium 0.6 mg/kg. Succinylcholine was deliberately avoided due to the reported association between EGPA and reduced plasma cholinesterase activity. Neuromuscular blockade was monitored using quantitative train-of-four (TOF) monitoring to ensure appropriate depth of paralysis and safe recovery. Tracheal intubation was performed using videolaryngoscopy (Glidescope) to minimize airway stimulation.

#### 3.8.3. Intraoperative Management and Cardiopulmonary Bypass

**Figure 2:**
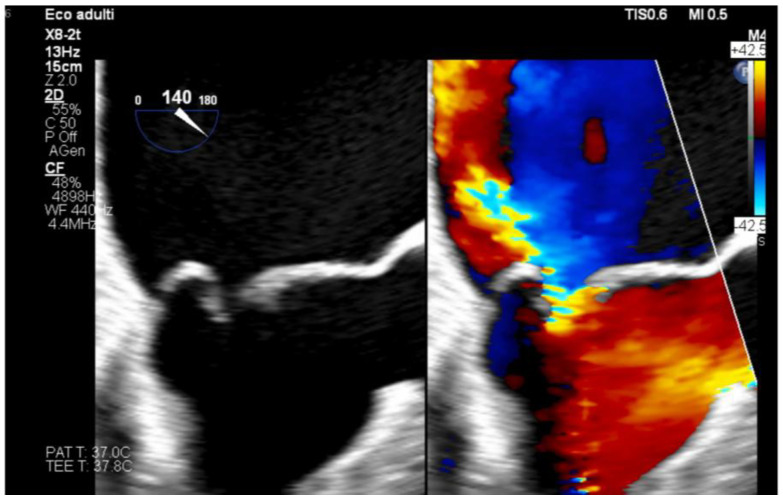
Preoperative transesophageal echocardiography from the illustrative case experience, demonstrating severe mitral regurgitation.

**Figure 3:**
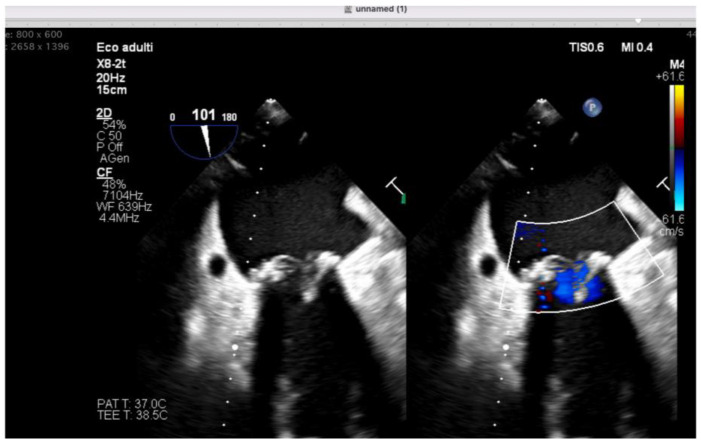
Transesophageal echocardiographic view after mitral valve replacement in the illustrative case, showing a normal function of the bioprosthetic valve without periprosthetic leak.

Anesthesia was maintained with sevoflurane (MAC 0.3), intravenous propofol, and sufentanil infusion. Following systemic heparinization, cardiopulmonary bypass was initiated, and mitral valve replacement was performed uneventfully. Transesophageal echocardiography confirmed severe mitral regurgitation pre-replacement and normal prosthetic valve function post-implantation without periprosthetic leak (Figure 2 and Figure 3). Cardiopulmonary bypass was successfully weaned with low-dose epinephrine infusion at 0.05 µg/kg/min, without significant hemodynamic instability.

#### 3.8.4. Postoperative Course and Analgesia

**Figure 4:**
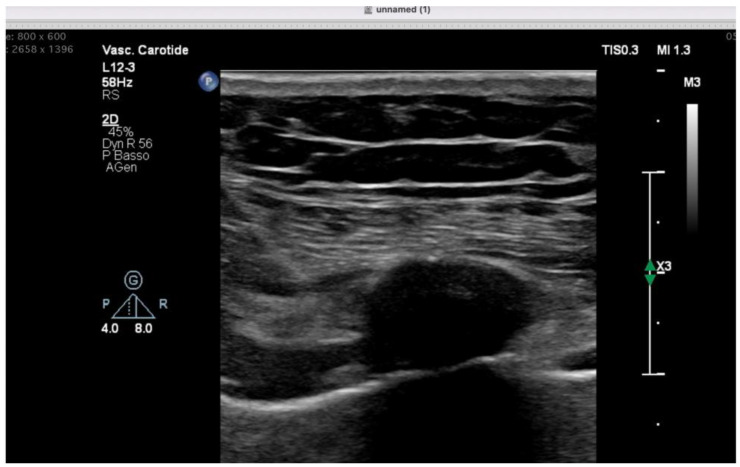
Ultrasound-guided parasternal block performed in the illustrative case as a part of an opioid-sparing analgesic strategy.

To minimize opioid exposure and reduce the risk of bronchospasm or respiratory depression, bilateral ultrasound-guided parasternal blocks were performed using 0.25% bupivacaine (10 mL per side) (Figure 4). Scheduled paracetamol 1000 mg every 8 h was administered. No systemic opioids were required postoperatively, and CPOT scores remained <1 throughout ICU stay. The patient was extubated 4 h after surgery and transferred to the cardiac surgery ward the following day. No bronchospastic episodes, allergic reactions, or cardiovascular complications occurred.

#### 3.8.5. Integrative Perspective

This case concretely illustrates the perioperative themes identified in the reviewed literature, namely airway protective strategies in severe asthma, avoidance of depolarizing neuromuscular blockade in the context of potential cholinesterase deficiency, phenotype-driven hemodynamic management in cardiac involvement, and opioid-sparing regional analgesia.

### 3.9. EGPA-Specific Perioperative Risk Profile

Although several anesthetic considerations in EGPA overlap with those applied in severe asthma, the available case literature suggests that EGPA introduces additional perioperative risk domains not fully captured by generic asthma management [48]. In particular, the coexistence of systemic vasculitis, potential myocardial involvement, chronic immunosuppression, and reported alterations in plasma cholinesterase activity creates a composite risk profile that differs from isolated obstructive airway disease. Cardiac manifestations, including myocarditis, ventricular dysfunction, and coronary involvement, may significantly influence hemodynamic management and intraoperative monitoring strategies. Chronic corticosteroid therapy and other immunosuppressive regimens introduce considerations related to adrenal suppression, infection risk, and wound healing that extend beyond asthma care. Furthermore, reports of reduced plasma cholinesterase activity and prolonged neuromuscular blockade highlight a potential pharmacologic vulnerability not typically encountered in asthma alone. These combined features support a phenotype-informed perioperative approach in EGPA, integrating airway protection, cardiovascular assessment, immunologic status, and neuromuscular pharmacology into anesthetic planning [9,17,34,49,50,51,52].

### 3.10. Personalized Medicine Perspective in EGPA Anesthetic Management

In EGPA, personalized perioperative management may extend beyond general clinical judgment and instead be anchored in disease phenotype, organ involvement, and immunologic profile (Table 3). EGPA is a heterogeneous condition, broadly characterized by overlapping eosinophilic-driven inflammation and systemic vasculitis. Approximately 30–40% of patients are ANCA positive, most commonly with myeloperoxidase (MPO) antibodies, while a substantial proportion are ANCA negative and may exhibit more prominent eosinophilic or cardiac manifestations [17].

This heterogeneity has practical anesthetic implications. ANCA-positive patients may demonstrate more overt vasculitic features, including peripheral neuropathy and renal involvement, whereas ANCA-negative or eosinophil-predominant phenotypes are more frequently associated with cardiac infiltration and myocardial dysfunction [4,17,52]. Preoperative evaluation may therefore integrate not only respiratory status but also cardiac imaging, biomarker assessment, and systemic organ involvement. Eosinophilia itself may reflect ongoing inflammatory activity and airway hyperreactivity, reinforcing the need for bronchoprotective induction strategies. In patients with documented myocardial involvement, invasive monitoring and cautious hemodynamic titration may be warranted [39,46]. Chronic corticosteroid therapy and biologic agents (e.g., anti-IL-5 therapies) further modify perioperative risk by influencing adrenal axis suppression, infection susceptibility, and inflammatory response. Additionally, reports of reduced plasma cholinesterase activity and prolonged neuromuscular blockade introduce a pharmacologic dimension to personalized care. Although not universally present, this potential enzymatic variability supports consideration of avoiding depolarizing neuromuscular blocking agents when feasible, the use of non-depolarizing alternatives, and quantitative neuromuscular monitoring to ensure safe recovery [9,33,42,52,53,54,55,56]. Taken together, these elements illustrate that precision in EGPA perioperative care is grounded in phenotype-informed risk stratification rather than solely in procedural customization. Integrating immunologic status, organ involvement, and pharmacologic susceptibility into anesthetic planning represents a clinically meaningful application of personalized medicine in this rare and heterogeneous disease.

The exclusive reliance on case-based evidence limits the certainty of proposed considerations and precludes the formulation of formal practice recommendations.

## 4. Conclusions

The available literature on perioperative anesthetic management in eosinophilic granulomatosis with polyangiitis is limited to isolated case reports and small descriptive series. Consequently, the evidence base remains low in methodological quality and highly heterogeneous. Despite these limitations, recurring perioperative themes emerge across published cases, including the need for careful airway management in patients with significant asthma, cautious selection of neuromuscular blocking agents, attention to potential cholinesterase variability, and consideration of cardiovascular involvement and chronic immunosuppressive therapy. The structured approach proposed in this manuscript may be considered as expert-informed, hypothesis-generating guidance derived from very low-quality evidence rather than formal recommendations. It is intended as a pragmatic synthesis of reported experiences to assist clinicians in managing complex perioperative scenarios in this rare disease. Further prospective data collection, collaborative multicenter reporting, and systematic perioperative documentation are needed to define better, more robust, evidence-based strategies for anesthetic management in EGPA and to validate the proposed framework.

## Figures and Tables

**Table 1:** Published case reports describing anesthetic or perioperative management in patients with EGPA (Churg–Strauss syndrome). ICU: intensive care unit; EGPA: eosinophilic granulomatosis with polyangiitis; GA: general anesthesia; ESP block: erector spinae plane block; NMBA: neuromuscular blocking agent; LMA: laryngeal mask airway; MVR: mitral valve replacement.

Author (Year)	Age/Sex	Surgical Context	EGPA Manifestations Relevant to Anesthesia	Chronic Therapy	Anesthetic Technique/ Perioperative Approach	Main Anesthetic Implications	Outcome
Im HS et al., 2010 [5]	34-year-old woman	Endoscopic sinus surgery	Severe asthma	Salmeterol 1 puff once a day	General anesthesia	Describes perioperative management in EGPA with attention to airway hyperreactivity and potential cholinesterase deficiency	Uneventful perioperative course; no reported bronchospasm or delayed recovery
Gerlach RM et al., 2013 [7]	58-year- old man	Endoscopic sinus surgery	Cardiac tamponade as the first presentation of EGPA	No chronic therapy (EGPA not previously diagnosed)	General anesthesia planned; focused echocardiography performed pre-induction	Highlights the importance of pre-surgery focused echocardiography in detecting occult cardiac involvement; underscores the risk of severe cardiovascular complications in previously unrecognized EGPA	Cardiac tamponade identified before surgery; surgery postponed; patient stabilized, and EGPA subsequently diagnosed and treated.
Taylor BL et al., 1990 [8]	64-year-old man 37-year-old woman	Perioperative/ICU context	Respiratory symptoms	Immunosuppressive therapy	Analysis of neuromuscular management	Describes reduced plasma cholinesterase activity in EGPA with potential for prolonged paralysis after succinylcholine; supports avoidance of depolarizing NMBAs unless enzyme activity is known	Prolonged neuromuscular blockade following succinylcholine; highlighted cholinesterase deficiency
Gurjar M et al., 2006 [9]	36-year-old woman	Modified radical mastectomy	Asthma, mononeuritis multiplex, steroid-induced diabetes, severe obstructive respiratory pattern	Inhaled budesonide 800 μg 12 hourly, salbutamol 200 μg 6 hourly; oral etophylline and theophylline; prednisolone 10 mg/day	Combined general anesthesia and thoracic epidural analgesia	Highlights multimodal strategy in EGPA with severe airway obstruction: continuation of asthma therapy, avoidance of NMBAs due to potential cholinesterase deficiency, use of LMA to reduce airway reactivity, thoracic epidural for analgesia, and potential reduction in bronchial hyperreactivity.	Uneventful intra- and postoperative course; stable respiratory and hemodynamic parameters.
Chung KY et al., 2009 [10]	22-year- old woman	Cholecystectomy	Asthma and systemic manifestation consistent with EGPA	Oral methylprednisolone 40 mg and salbutamol 1 g inhalation	General anesthesia	Highlights perioperative management considerations in EGPA, including airway hyperreactivity, need for perioperative steroid coverage, and cautious use of neuromuscular blocking agents given potential cholinesterase deficiency	Uneventful perioperative course with appropriate steroid supplementation and careful anesthetic management.
Chia PH, 2018 [11]	41-year-old woman	Hysterectomy and salpingo-oophorectomy	Asthma and pansinusitis	Fluctuating doses of prednisolone, mycophenolate, and bronchodilators	General anesthesia with rocuronium and sugammadex reversal	Highlights the role of sugammadex for reliable NMBA reversal in patients where cholinesterase deficiency is a concern	Successful reversal with sugammadex; no prolonged paralysis or respiratory compromise
Mohamed H et al., 2021 [12]	58-year-old woman	Intramedullary femoral nailing	Asthma, recurrent lower respiratory tract infections, and sinusitis	Salbutamol inhaler 100 μg, long-term anticholinergic inhaler 2.5 mg, formoterol fumarate/fluticasone propionate inhaler 10 μg, and prednisolone 5 mg/day	Spinal anesthesia and peripheral nerve block	Preference for regional techniques to avoid airway manipulation in EGPA with pulmonary involvement	Stable intraoperative course; no perioperative respiratory complications
Melemeni A et al., 2020 [13]	15-year-old woman	Peri-induction event: esophagomyotomy	Asthma and upper respiratory tract infections	Budesonide/formoterol (160 + 4.5 μg) and montelukast sodium 10 mg	General anesthesia induction	Describe severe anaphylactic shock after induction as the first presentation of EGPA; stress vigilance for allergic reactions	Severe peri-induction anaphylactic shock; EGPA diagnosed following the event
Barbosa H et al., 2023 [14]	41-year-old man	Ambulatory/day surgery	Chronic sinusitis	Systemic and inhaled steroids (50 μg salmeterol, 500 μg fluticasone propionate, 20 μg ipratropium bromide, and mepolizumab injection once a month	Regional anesthesia	Demonstrates the feasibility and safety of opioid-sparing regional techniques in EGPA.	Successful ambulatory procedure with opioid-sparing regional anesthesia; no complications reported
Atlapure B et al., 2025 [15]	6-year-old girl	Pediatric thoracoscopic biopsy	Not specified	Not specified	General anesthesia with tailored airway strategy and ESP block	Highlights individualized perioperative management in complex pediatric EGPA	Successful perioperative management in a pediatric patient; no major complications reported
This work (Torre and Pirri)	64-year-old man	Cardiac surgery (MVR)	Asthma with recurrent bronchospastic episodes, chronic rhinosinusitis, moderate obstructive ventilatory deficit	Prednisone 10 mg/day and budesonide-formoterol inhalational therapy	General anesthesia and parasternal block	Opioid-sparing strategy, avoidance of succinylcholine, videolaryngoscopy	Extubation at 4 h postoperatively; no bronchospasm, allergic reactions, or cardiovascular instability; uneventful recovery.

**Table 2:** Key anesthetic considerations in eosinophilic granulomatosis with polyangiitis (EGPA). TOF: quantitative train-of-four monitoring; HPA: hypothalamic–pituitary–adrenal.

Domain	EGPA-Related Risk	Anesthetic Considerations
Airway	Severe asthma, eosinophilic inflammation	Preoperative optimization, avoidance of airway irritation, and bronchodilatory agents
Induction	Bronchospasm, hemodynamic instability	Etomidate, ketamine, and avoidance of histamine-releasing drugs
Maintenance	Airway hyperreactivity	Sevoflurane-based balanced anesthesia
Neuromuscular blockade	Possible cholinesterase deficiency	Avoid succinylcholine when feasible, prefer non-depolarizing agents with quantitative TOF and planned reversal (sugammadex)
Analgesia	Risk of bronchospasm, respiratory depression	Opioid-sparing strategies, regional techniques
Steroids	Chronic corticosteroid therapy	Individualized perioperative supplementation based on HPA axis risk

**Table 3:** Phenotype-informed perioperative considerations in EGPA. ICU: intensive care unit; HPA: hypothalamic–pituitary–adrenal.

Domain	EGPA-Related Risk Profile	Preoperative Considerations	Intraoperative Considerations	Postoperative Considerations	Evidence Level
Airway/respiratory	Severe asthma, airway hyperreactivity, eosinophilic inflammation	Assess asthma control; consider bronchodilator optimization; evaluate recent exacerbations	Bronchoprotective induction may be considered; avoid histamine-releasing drugs when feasible; ensure adequate anesthetic depth during airway manipulation	Close monitoring for bronchospasm; early bronchodilator therapy if needed	Case reports
Cardiovascular (myocardial involvement)	Myocardial infiltration: cardiomyopathy, heart failure, arrhythmias	Consider echocardiography and biomarkers when clinically indicated	Hemodynamic monitoring tailored to cardiac phenotype; cautious fluid and vasoactive titration	Surveillance for arrhythmias and ventricular dysfunction	Case reports
Coagulation/vasculitis activity	Active systemic vasculitis; potential endothelial dysfunction	Assess disease activity; review immunosuppressive therapy; consider inflammatory markers	Maintain hemodynamic stability; minimize endothelial stress	Monitor for thrombotic or inflammatory complications	Descriptive literature
Steroid therapy/Adrenal axis	Chronic corticosteroid exposure; potential adrenal suppression	Evaluate chronic corticosteroid exposure and risk of HPA axis suppression	Perioperative supplementation may be individualized according to chronic dose and surgical stress	Monitor glycemic control, infection risk	Case-based evidence; endocrine consensus extrapolation
Biologic therapy	Immunomodulation; infection susceptibility	Review biologic timing; assess infection risk	No specific intraoperative modification is typically required; maintain aseptic vigilance	Monitor wound healing and infection	Extrapolated evidence
Neuromuscular pharmacology	Possible reduced plasma cholinesterase activity (reported variability)	Review prior anesthetic history; consider baseline risk factors	Consider avoiding depolarizing neuromuscular blockers when feasible; use quantitative neuromuscular monitoring	Confirm full recovery before extubation	Case reports
Postoperative surveillance	Respiratory instability; cardiac complications; immunosuppression	Risk-stratified postoperative disposition planning		Consider high-level monitoring (e.g., ICU) in severe phenotypes	Case-based evidence

## Data Availability

No new data were created or analyzed in this study. Data sharing is not applicable to this article.

## Abbreviations

The following abbreviations are used in this manuscript:

EGPA	Eosinophilic granulomatosis with polyangiitis
CS	Churg–Strauss
NSAIDs	Nonsteroidal anti-inflammatory drugs
ACR	American College of Rheumatology
